# Effects of automated peritoneal dialysis and continuous ambulatory peritoneal dialysis on residual kidney function in Chinese patients with new-onset end stage kidney disease

**DOI:** 10.1080/0886022X.2026.2735546

**Published:** 2026-09-27

**Authors:** Lijun Sun, Mengna Ruan, Ziyu Liu, Xiaojing Tang, Lin Li, Senyan Liu

**Affiliations:** a Department of Nephrology and Endocrinology, Shanghai 411 Hospital, China RongTong Medical Healthcare Group Co. Ltd., Shanghai, China; b Department of Nephrology, Changzheng Hospital, Naval Medical University, Shanghai, China

**Keywords:** Residual kidney function, automated peritoneal dialysis, continuous ambulatory peritoneal dialysis, end-stage kidney disease

## Abstract

Automated peritoneal dialysis (APD) is a widely used dialysis modality for patients with end-stage kidney disease (ESKD). However, controversy remains regarding the rate of residual kidney function (RKF) decline between patients treated with APD and continuous ambulatory peritoneal dialysis (CAPD). This study aimed to investigate the effect of peritoneal dialysis modality on the rate of RKF decline in Chinese patients with new-onset ESKD. We conducted a single-center retrospective study to explore the association between PD modalities and RKF decline. A total of 53 patients with new-onset ESKD were enrolled. Of these, 19 patients started with APD and 34 with CAPD. The rates of RKF decline were compared between two groups. Multivariate linear regression was performed to identify risk factors for rapid decline of RKF. Baseline RKF and urine output were comparable between the APD and CAPD groups. At 6 months after PD initiation, RKF was 1.9 ± 1.6 mL/min/1.73 m^2^ in the APD group, significantly lower than 3.0 ± 1.7 mL/min/1.73 m^2^ in the CAPD group (*p* = 0.043). The APD group had a significantly faster mean rate of RKF decline (−6.6 ± 2.5 vs. −4.3 ± 2.3 mL/min/1.73 m^2^/year, *p* = 0.002). In multivariate linear regression adjusted for established risk factors, APD use (vs. CAPD) (β = 0.93; *p* = 0.004) and baseline RKF (β = 0.38; *p* < 0.001) were significantly associated with accelerated RKF decline. Our findings indicate that Chinese patients initiating dialysis with APD experience faster RKF decline within the first 6 months compared with those starting PD with CAPD. Furthermore, high baseline RKF is associated with rapid RKF decline.

## Introduction

The prevalence of end-stage kidney disease (ESKD) is rising rapidly in China. According to the China Renal Data System (CNRDS), the number of patients receiving dialysis in mainland China increased from 235,000 in 2011 to 1.183 million in 2024. Of these, approximately 156,000 were treated with peritoneal dialysis (PD). As a key component of comprehensive kidney replacement therapy (KRT), PD offers substantial benefits for patients with ESKD, including better preservation of vascular access and residual kidney function (RKF), fewer center visits, and lower transmission risk of infectious diseases such as respiratory viruses and blood-borne viruses [1].

Two PD modalities are available: automated peritoneal dialysis (APD) and continuous ambulatory peritoneal dialysis (CAPD). Driven by its favorable lifestyle profile and reliable achievement of dialysis adequacy and ultrafiltration [2], the utilization of APD has increased markedly in recent years. In the United States, the proportion of PD patients on APD rose from 47% in 2000 to 80% in 2015 [3], and 40%–60% of PD patients in most European countries received APD [4]. Owing to higher costs, APD use is lower in developing countries than in developed nations (15.8% vs. 42.4% of PD patients, respectively) [5]. A large Chinese observational study following 100,351 patients between 2005 and 2015 reported that only 368 ever received APD [6].

The prognostic significance of RKF in dialysis patients has been well recognized. Previous studies have demonstrated that PD, compared with hemodialysis, is associated with better RKF preservation [7]. However, controversy remains regarding whether APD or CAPD offers superior RKF protection. Some studies have suggested more rapid RKF decline in patients treated with APD [8–12]. To our knowledge, no comparable studies have been conducted in the Chinese population. We therefore performed this study to investigate whether patients initiating dialysis with APD experience a faster rate of RKF decline than those starting with CAPD.

## Materials and methods

### Study population

We conducted a single-center, retrospective study of prospectively collected data at Peritoneal Dialysis Unit of Changzheng Hospital, Shanghai, China. This retrospective study was approved by the Ethics Board of Changzheng Hospital, and the requirement for written informed consent was waived due to the anonymized and non‑interventional nature of the analysis (Ethics approval number: CZ2024-028). The study was designed in accordance with the Declaration of Helsinki. All patients starting PD between January 1, 2023, and December 31, 2023, who fulfilled the following inclusion criteria were eligible for the study: (i) Age older than 18 years; (ii) Minimum follow-up of 6 months on PD; (iii) Baseline 24-h urine volume ≥ 400 mL/24 h and GFR ≥ 2 mL/min/1.73m^2^. Exclusion criteria were: (i) prior kidney replacement therapy (i.e. a patient who transitioned to PD from a failing kidney allograft or from chronic HD); (ii) incomplete data for study; (iii) Any alteration to their everyday peritoneal dialysis prescription throughout the study period (including incremental peritoneal dialysis).

### Dialysis prescription

APD patients received 5 exchanges during an 10-h nighttime dwell with 2.0 L of instilled volume, using a cycler (Baxter 5C6M00, Baxter healthcare SA, Switzerland). CAPD patients received four exchanges per day, routinely with 2.0 L dialysate. All patients were treated with low-calcium dialysate containing 1.5% glucose (Baxter Healthcare [Guangzhou] Co., Ltd., Guangzhou, China). In the present study, all patients were prescribed to achieve the target of weekly Kt/V urea >1.7, which is the minimal target of dialysis dose for PD patients.

### Data collection

Collected data encompassed patient demographic characteristics, including age at PD initiation, sex, height, weight, primary etiology of ESKD, comorbidities (e.g. diabetes mellitus, hypertension), and PD modality (CAPD or APD). Baseline laboratory parameters obtained at PD commencement comprised hemoglobin, albumin, calcium, phosphorus, intact parathyroid hormone (iPTH), C‑reactive protein (CRP), and peritoneal equilibration test (PET) parameters (corrected dialysate‑to‑plasma creatinine ratio), all determined *via* standardized laboratory protocols. Additional clinical data incorporated blood pressure readings, prescribed medications (e.g. angiotensin‑converting enzyme inhibitors [ACEI], angiotensin receptor blockers [ARB], diuretics), and episodes of peritonitis.

Residual kidney function (RKF) was evaluated by 24‑hour urine volume and estimated glomerular filtration rate (GFR). During the predialysis phase, GFR was calculated using the 2021 CKD‑EPI equation [13]. Following PD initiation, residual GFR (rGFR) was computed as the mean of 24‑hour urinary creatinine clearance (Ccr) and urea clearance, adjusted for body surface area (mL/min/1.73 m^2^) [14]. All patients underwent one assessment of 24‑hour urine volume and GFR within 1 week prior to PD initiation, with two subsequent assessments performed at 3 and 6 months post‑PD initiation, respectively.

### Statistical analysis

Categorical variables were presented as percentages, normally distributed continuous variables as mean ± SD, and non-normally distributed continuous variables as median (range). Baseline characteristics were compared using one-way ANOVA for continuous variables and χ^2^ test for categorical variables. Pearson correlation analysis was used to explore associations between RKF decline rate and other variables. Multivariate linear regression was performed to identify independent risk factors for RKF decline. A *P* value < 0.05 was considered statistically significant. All analyses were conducted using SPSS 25.0 (SPSS Inc., Chicago, IL, USA).

## Results

### Patient characteristics

Baseline demographic, clinical and biochemical characteristics of the enrolled patients are summarized in Table 1. No significant differences were observed between the APD and CAPD groups in terms of age, gender, body mass index (BMI), mean arterial pressure, residual glomerular filtration rate (rGFR), urine volume, weekly urea Kt/V, 4-h D/P creatinine ratio (4-h D/P cr), and other biochemical indicators. In addition, the 6-month cumulative incidence of peritonitis was comparable between the two groups, with no statistically significant difference.

**Table 1.** Baseline characteristics.

Variable	CAPD (*n* = 34)	APD (*n* = 19)	*p* value
Sex (men)	21 (62%)	16 (82.4%)	0.123
Age (years)	53.1 ± 14.3	49.0 ± 14.3	0.424
Primary kidney disease			
Glomerulonephritis	20 (58.8%)	12 (63.2)	0.964
Diabetes mellitus	7 (20.5%)	3 (15.8%)	
Renal vascular disease	4 (11.8%)	2 (10.5%)	
Other	3 (8.8%)	2 (10.5%)	
Systolic blood pressure (mmHg)	139.02 ± 21.7	132.5 ± 25.7	0.329
Diastolic blood pressure (mmHg)	89.3 ± 13.6	83.6 ± 12.6	0.305
Use of RAS blockers	12 (35.3%)	8 (42.1%)	0.624
Hemoglobin (g/dL)	10.6 ± 2.0	10.2 ± 1.9	0.869
Albumin (g/dL)	3.5 ± 0.5	3.6 ± 0.3	0.250
Blood urea nitrogen (mg/dL)	50.1 ± 10.9	52.3 ± 14.1	0.533
Creatinine (mg/dL)	7.3 ± 2.8	7.7 ± 3.1	0.866
Body mass index (kg/m^2^)	22.4 ± 2.9	22.7 ± 3.0	0.963
Urine production at start of dialysis (L/24 h)	0.97 ± 0.44	0.89 ± 0.29	0.471
rGFR at start of dialysis (mL/min per 1.73 m^2^)	5.1 ± 2.3	5.2 ± 1.8	0.878
Weekly Kt/V urea	1.98 ± 0.16	2.06 ± 0.18	0.087
4-hr dialysate-to-plasma creatinine ratio	0.72 ± 0.11	0.71 ± 0.08	0.904
Peritonitis rate (episodes/patient-years)	0.18	0.11	0.622

PD, peritoneal dialysis; CAPD, continuous ambulatory peritoneal dialysis; APD, automated peritoneal dialysis; RAS, renin-angiotensin system.

Data are presented as mean ± SD or as numbers (percentages).

### Decline of residual kidney function

As shown in Figure 1a, residual kidney function (RKF) at 6 months after peritoneal dialysis (PD) initiation was 3.0 ± 1.7 mL/min/1.73 m^2^ in the CAPD group, which was significantly higher than that in the APD group (1.9 ± 1.6 mL/min/1.73 m^2^, *p* = 0.043). The annual decline rate of residual kidney function (RKF) differed significantly between the two groups (APD vs. CAPD: −6.6 ± 2.5 vs. −4.3 ± 2.3 mL/min/1.73 m^2^/year, *p* = 0.002). At the 6-month follow-up, complete loss of RKF occurred in 3 patients (9%) in the CAPD group and 3 patients (16%) in the APD group, with no significant intergroup difference (*p* = 0.655). Figure 1b demonstrates that the APD group presented a faster reduction in urine volume from baseline to 6 months after PD commencement. At 6 months, the mean daily urine volume was 0.30 ± 0.19 L/d in the APD group and 0.59 ± 0.36 L/d in the CAPD group, with a statistically significant difference between groups (*p* = 0.003).

**Figure 1.**
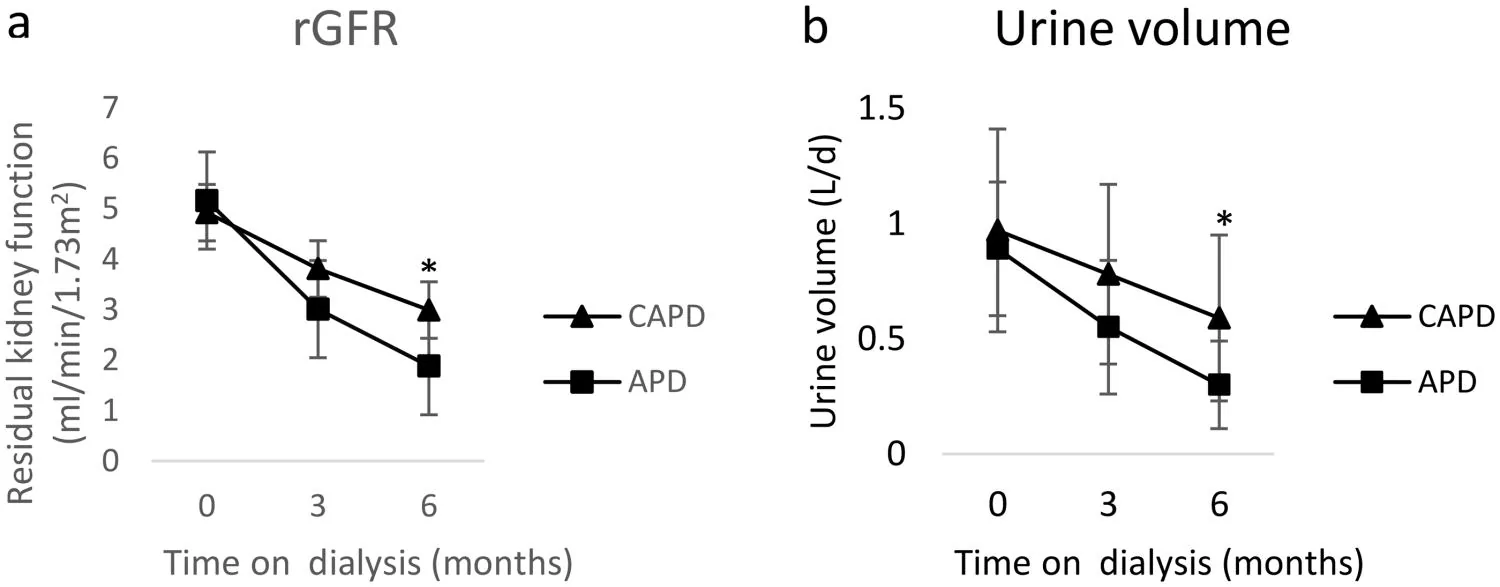
Comparison of the decline in residual kidney function (a) and urine output (b) between the CAPD group and APD group. * *p* < 0.05 vs. APD group. CAPD, continuous ambulatory peritoneal dialysis; APD, automated peritoneal dialysis.

### Predictors of fast decline of residual kidney function

Multivariate linear regression analysis was performed to explore independent factors associated with accelerated RKF decline, incorporating established risk variables including age, sex, diabetes mellitus, BMI, serum albumin, baseline residual kidney function, baseline urine output, and dialysis modality (APD versus CAPD). The results demonstrated that APD modality and higher baseline RKF were marginally correlated with a more rapid reduction in residual kidney function (Table 2).

**Table 2.** Multiple linear regression analysis of variables affecting the decline rate of RKF.

Variables	β	95%CI	*p* value
Age (years)	−0.02	−0.04 to 0.01	0.249
Male (vs. female)	0.06	−0.56 to 0.69	0.844
DM (versus non-DM)	0.28	−0.44 to 1.01	0.429
Body mass index (kg/m^2^)	0.04	−0.06 to 0.15	0.413
Albumin (g/dL)	0.05	−0.55 to 0.65	0.858
Baseline RKF (mL/min/1.73 m²)	0.38	0.24 to 0.52	<0.001
Baseline urine volume (L/day)	−0.59	−1.37 to 0.19	0.136
APD vs. CAPD	0.93	0.32 to 1.55	0.004

RKF, residual kidney function; DM, diabetes mellitus; APD, automated peritoneal dialysis; CAPD, continuous ambulatory peritoneal dialysis.

## Discussion

This study compared the decline in RKF between APD and CAPD among patients with newly diagnosed ESKD initiating PD. For the first time, we found that Chinese ESKD patients who commenced PD with APD exhibited a higher risk of early RKF loss within the first 6 months of dialysis initiation. This accelerated RKF decline was more evident in patients with a higher baseline residual glomerular filtration rate (rGFR). Although the incidence of anuria at 6 months was numerically higher in the APD group (16% versus 9% in the CAPD group), the difference did not reach statistical significance, likely owing to limited sample size. This trend should be interpreted cautiously. Our finding that APD is associated with more rapid RKF deterioration compared with CAPD is consistent with several previous investigations [8–12]. In contrast, other studies have reported no significant difference in the rate of RKF decline between the two PD modalities [15–17].

RKF is closely associated with superior survival, reduced morbidity, and enhanced quality of life in patients receiving either peritoneal dialysis or hemodialysis [18,19]. Accumulating clinical evidence and meta-analyses have confirmed that PD confers superior RKF preservation compared with conventional hemodialysis [20], which is attributed to the favorable hemodynamic stability, absence of extracorporeal circulation-related inflammation, and continuous, gentle ultrafiltration unique to PD. The proportion of patients initiating PD with APD has increased steadily in recent years, given its multiple advantages over CAPD, such as a lower peritonitis risk, improved small solute clearance, alleviated back pain, and favorable psychosocial outcomes. Considering the well-documented accelerated loss of RKF in APD recipients, individualized and careful dialysis modality selection is therefore essential for patients new to peritoneal dialysis. In 2020, the International Society for Peritoneal Dialysis (ISPD) recommended incremental peritoneal dialysis (IPD) to optimize patient experience and deliver high-quality, goal-directed PD care [21]. Although several studies have indicated that IPD may facilitate residual kidney function preservation, the available evidence remains inconsistent [22–24]. Future studies should further clarify whether incremental APD confers superior preservation of RKF.

Multiple APD modalities are currently available, including continuous cyclical peritoneal dialysis (CCPD), intermittent peritoneal dialysis (IPD), nightly intermittent peritoneal dialysis (NIPD), and tidal peritoneal dialysis (TPD). Two studies have reported that intermittent APD accelerate RKF decline [8,9]. Hiroshige et al. confirmed a pronounced reduction in RKF among patients receiving NIPD and CCPD [8]. Consistently, our study observed rapid RKF loss in all participants treated with NIPD, which supports these earlier findings. Adachi et al. demonstrated that tidal APD provides superior RKF preservation compared with non‑tidal APD [25]. Further investigations are required to clarify the differential impacts of distinct APD modalities on RKF preservation.

The mechanisms responsible for accelerated RKF decline in patients receiving APD remain incompletely elucidated. As mentioned in the foregoing studies [8–10], acute fluctuations in volume and osmotic load induced by nocturnal dialysis might cause kidney injury, and the overlap between the nocturnal ultrafiltration period and the circadian blood‑pressure nadir further aggravated the impairment. In addition, Hiroshige et al. demonstrated that the rate of RRF decline in patients undergoing APD was comparable to that observed in hemodialysis patients. It is therefore hypothesized that the intermittent nature of the therapy itself, rather than the dose of solute and fluid removal, plays a pivotal role in the loss of residual renal function. Selby et al. confirmed that APD exerts profound influences on systemic hemodynamics. Compared with CAPD, APD involves more frequent fill–drain cycles, accompanied by progressive elevation in peripheral vascular resistance and reduced cardiac output, which may be partially attributed to body temperature reduction during overnight dialysis [26,27]. All these hemodynamic disturbances could compromise renal perfusion and consequently accelerate residual kidney function deterioration.

Priyanka et al. reported that early icodextrin use preserved RKF and improves blood pressure control versus glucose dialysate in pediatric peritoneal dialysis patients [28]. A meta-analysis demonstrated that the use of neutral-pH, low-glucose degradation products solutions resulted in better preservation of residual renal function and greater urine volumes [29]. Nevertheless, these two novel dialysates have been commercially available in China for only a short period, resulting in limited local clinical experience. With the growing clinical use of these solutions, more relevant clinical evidence will be accumulated in the future.

Previous studies showed that factors predicting the loss of residual solute clearance and urine output were different [30]. Proteinuria, baseline residual GFR, and the use of diuretics were independently related to the rate of RKF decline in CAPD patients, while proteinuria, glucose exposure, and the number of peritonitis episodes were independent predictors for the development of anuria. Our study suggested that APD selection and higher baseline RKF were associated with rapid decline of RKF, which is in line with previous studies. Patients with higher baseline RKF may have glomeruli under high-pressure, high-flow states; the abrupt hemodynamic shift after dialysis initiation (reduced volume overload, RAAS blockade) may lead to rapid normalization of hyperfiltration, manifesting as a steep early drop in measured RKF (regression-to-the-mean effect). Other factors analyzed in our study were not associated with the rapid decline in RKF.

Our study does have several limitations. First, As this was a retrospective observational study, the nonrandom allocation of APD and CAPD based on clinical judgment may introduce significant selection bias. Second, the relatively small sample size precluded subgroup analyses stratified by primary renal disease. Diabetes is known to exert a detrimental impact on residual kidney function preservation in patients with ESKD; thus, the rate of RKF decline may differ substantially between diabetic and non-diabetic individuals with ESKD. Third, the short follow-up period limited our ability to evaluate the long-term outcomes of different dialysis modalities. Finally, he relatively small sample size may increase the risk of overfitting in the multivariate regression analysis. Therefore, the conclusions of the present study should be interpreted with caution. Large-sample, multicenter prospective studies are required in the future to further verify our findings and establish more robust predictive regression models.

## Conclusion

In summary, the present study suggested that initiation of peritoneal dialysis with APD (NIPD) is independently associated with more rapid RKF decline and a higher risk of subsequent anuria, compared with CAPD. Higher baseline GFR was also associated with accelerated RKF loss. Our findings suggest that CAPD may be preferable for incident PD patients, whereas APD should be reserved for those with high peritoneal membrane transport status or specific psychosocial demands. Notably, all patients received standard glucose dialysate, and APD was exclusively NIPD. Thus, our results cannot be generalized to other dialysates or alternative APD regimens. Large-scale randomized controlled trials are further required to clarify the comparative effects of APD and CAPD on long-term RKF preservation and guide individualized clinical decision-making.

## Data Availability

The datasets used and/or analyzed during the current study are available from the corresponding author on reasonable request.
